# A Novel Score for mHealth Apps to Predict and Prevent Mortality: Further Validation and Adaptation to the US Population Using the US National Health and Nutrition Examination Survey Data Set

**DOI:** 10.2196/36787

**Published:** 2022-06-14

**Authors:** Shatha Elnakib, Andres I Vecino-Ortiz, Dustin G Gibson, Smisha Agarwal, Antonio J Trujillo, Yifan Zhu, Alain B Labrique

**Affiliations:** 1 Department of International Health Johns Hopkins Bloomberg School of Public Health Baltimore, MD United States

**Keywords:** C-Score, validation, mortality, predictive models, mobile phone

## Abstract

**Background:**

The *C-Score*, which is an individual health score, is based on a predictive model validated in the UK and US populations. It was designed to serve as an individualized *point-in-time* health assessment tool that could be integrated into clinical counseling or consumer-facing digital health tools to encourage lifestyle modifications that reduce the risk of premature death.

**Objective:**

Our study aimed to conduct an external validation of the C-Score in the US population and expand the original score to improve its predictive capabilities in the US population. The C-Score is intended for mobile health apps on wearable devices.

**Methods:**

We conducted a literature review to identify relevant variables that were missing in the original C-Score. Subsequently, we used data from the 2005 to 2014 US National Health and Nutrition Examination Survey (NHANES; N=21,015) to test the capacity of the model to predict all-cause mortality. We used NHANES III data from 1988 to 1994 (N=1440) to conduct an external validation of the test. Only participants with complete data were included in this study. Discrimination and calibration tests were conducted to assess the operational characteristics of the adapted C-Score from receiver operating curves and a design-based goodness-of-fit test.

**Results:**

Higher C-Scores were associated with reduced odds of all-cause mortality (odds ratio 0.96, *P*<.001). We found a good fit of the C-Score for all-cause mortality with an area under the curve (AUC) of 0.72. Among participants aged between 40 and 69 years, C-Score models had a good fit for all-cause mortality and an AUC >0.72. A sensitivity analysis using NHANES III data (1988-1994) was performed, yielding similar results. The inclusion of sociodemographic and clinical variables in the basic C-Score increased the AUCs from 0.72 (95% CI 0.71-0.73) to 0.87 (95% CI 0.85-0.88).

**Conclusions:**

Our study shows that this digital biomarker, the C-Score, has good capabilities to predict all-cause mortality in the general US population. An expanded health score can predict 87% of the mortality in the US population. This model can be used as an instrument to assess individual mortality risk and as a counseling tool to motivate behavior changes and lifestyle modifications.

## Introduction

### Background

In the United States, 60% of all adults have at least one chronic condition, and 42% have >1 [1,2], leading to >1.7 million deaths annually [3]. Reliable indicators of current and future health can be integrated into digitally enabled strategies to modify behaviors and reduce the risk of adverse outcomes and death. Therefore, there is a growing demand for evidence-based tools, supported by ubiquitous innovations such as wearable technologies, that could help clinicians and individuals to calculate the risk of disease and predict future health outcomes [4,5]. Such tools and technologies often collect data on risk factors that can be integrated into an index to provide information on current and future disease risks. The advent of wearable technologies and other readily accessible nonclinical sources of anthropometric or biometric data has challenged us to evaluate the value of extending classical metrics to achieve greater precision and predictive accuracy. When accurate, such tools have tremendous potential to inform lifestyle improvements and drive sustained changes in modifiable risk factors that can enhance health status.

In recent years, a number of risk-scoring algorithms and models have demonstrated the capacity to predict adverse health outcomes such as the risk of developing cardiovascular disease [6], diabetes [7], hypertension [8], and very specific cancers [9] and predict complications following surgery [10]. However, existing models or applications are often reserved for use by clinicians or incorporate the mathematical analysis of data points that require invasive testing (eg, blood tests). These models are rarely presented in friendly digital formats or provide advice to clients on specific modifiable behaviors. In addition, most prognostic indices have primarily focused on predicting short-term mortality among older adults and high-risk individuals, whereas fewer indices have focused on prognostic health assessment of the general population [11-16].

The C-Score, derived from metrics that are easily reported by a person and augmented by measures derivable from most smartphones, is designed as a tool for individualized health risk prediction and can be used as a basis for directing targeted lifestyle modifications to reduce the risk of future adverse outcomes. Clift et al [17] developed and validated the C-Score model using a prospective cohort analysis, leveraging the UK Biobank data set [17]. They found that the C-Score had good predictive capabilities for all-cause mortality within 10 years for adults aged between 40 and 69 years. The points-based model had good discrimination with a c-statistic of 0.66, and a Cox model with the C-Score and age had improved discrimination (c-statistic 0.74) and good calibration. Although the UK Biobank data set is an unparalleled resource of extensive health information with >400 peer-reviewed publications to date, its sampling population is volunteer based and hence not entirely representative of the UK population [18]. Keyes et al [19] articulated several concerns related to the nonrepresentativeness of this sample population, whereas Batty et al [20] concluded that risk factor associations in the UK Biobank seem to be generalizable, after comparing with pooled data from the Health Surveys for England and the Scottish Health Surveys.

### Objective

In this study, we conducted an external validation of the C-Score in the US population and expanded the original score to improve its predictive capabilities in the US population [17]. The C-Score is a mobile health app that can be used on wearable devices.

For the external validation, we assessed the discrimination and calibration of the original C-Score in the US population using the US National Health and Nutrition Examination Survey (NHANES). For the expansion and adaptation of the model, we reviewed the literature and tested additional predictors of all-cause mortality in the US population to improve the predictive capacity of the model.

## Methods

### The C-Score

The risk models were developed following an extensive literature review that identified key risk factors for all-cause mortality [17]. The review yielded eight key predictor variables: age, cigarette consumption, alcohol consumption, sleeping duration, self-rated health, waist to height (WtHR) ratio, resting heart rate, and reaction time. Given the interest in modifiable risk factors, age was not included in the calculation of the score. Relative weightings, which were developed by Clift et al [17], using hazard ratios extracted from each identified study, were used to generate a points-based score. The lowest risk was denoted with a 0, with increases in scores indicating higher than optimal risk. The overall score totaled 25 points and was multiplied by 4 to generate a sum of 100 (Table 1). The score operates in a penalizing fashion, with users starting with 100 points and losing points for each health domain in accordance with the hazard ratio extracted from the literature. Thus, the C-Score is an evidence-based consolidated index that uses 7 parameters to predict mortality. The points-based C-Score model performed moderately well in the United Kingdom with an area under the curve (AUC) >0.66 and high calibration [17]. More detailed information on the development of the score can be found elsewhere [17].

**Table 1:** Points-based score assigned to each explanatory variable for the original C-Score model.^a^

C-Score input	Points assigned, range
Resting heart rate (beats per minute)	0-7.83
Average hours of sleep per night	0-10.26
Waist to height ratio	0-10.8
Self-rated health (ordinal scale: excellent, good, fair, and poor)	0-31.32
Cigarette smoking (status and cigarettes per day)	0-12.96
Alcohol consumption (units per week)	0-19.44
Reaction time	0-6.75

^a^The reaction time variable is not present in the main National Health and Nutrition Examination Survey sample. Therefore, we did not include this in the main analysis. For the sensitivity analysis, we did not include alcohol consumption or sleep duration as these variables were not present in the National Health and Nutrition Examination Survey III.

### Data Source and Validation Population

The NHANES is a large cross-sectional population-based survey that combines interviews with physical examinations, thereby serving as a rich source of both self-reported and directly measured biometric data. Each survey round includes a nationally representative sample of approximately 5000 individuals and is conducted regularly. The NHANES questionnaire elicits information pertaining to sociodemographic, dietary, physical, and health-related characteristics. Details of the NHANES study design have been described in previous studies [21,22]. To validate the C-Score, we pooled the NHANES survey data from 2005 to 2014, resulting in data from 28,078 participants.

As mortality data are not readily collected as part of the NHANES, the National Center for Health Statistics has matched 1999 to 2014 data with death certificate records from the National Death Index (NDI), which have been made available for public use. Mortality ascertainment was based on a probabilistic match between the NHANES and NDI death certificate records. These data were, in turn, linked with NDI mortality data using participants’ social security number, first name, middle initial name, last name or father’s surname, month of birth, day of birth, year of birth, state of birth, state of residence, race, and sex, yielding a sample of 28,033 participants with complete information on mortality. The methodology for the data linkage has been described in detail by the National Center for Health Statistics [23].

We linked the anonymized NHANES survey data with the anonymized NDI mortality data, which included mortality follow-up data from December 31, 2015. The matching yielded a sample of 28,033 participants. This was the sample for which the external validation of the C-Score was conducted. It was also the sample for which the C-Score model was adapted and expanded to improve its performance in the US population.

Following the development of the adapted model, we conducted another round of validation as a sensitivity analysis, using data obtained from NHANES III, a survey conducted from 1988 to 1994, which included the mortality data of 6591 participants. The NHANES III data analysis missed 2 of the 7 variables included in the risk model (sleep duration and alcohol consumption); therefore, the C-Score was calculated in the absence of these risk factors.

### Predictor Variables

The explanatory variables in this study were extracted from the questionnaire data and examination data from the 5 NHANES waves. The questionnaire data included age (in years), cigarette consumption (average number of cigarettes per day), alcohol consumption (average number of alcoholic drinks per week), and sleep duration (hours per day). Self-rated health was transformed from a 5-point scale (from poor to excellent) into a 4-point scale in which *excellent* and *very good* health were merged into one category to better match with the UK Biobank variable. The NHANES examination data were collected by trained health technicians, and information was collected on WtHR (waist circumference divided by height) and resting heart rate (beats per minute). Reaction time was missing from the 2005 to 2014 NHANES data but was measured as part of a computerized Neurobehavioral Evaluation System 2.

### Expanding the Set of Variables for the Original C-Score Model

We conducted a subsequent literature review of predictors of all-cause mortality in the United States and identified a set of clinical factors and sociodemographic variables for which there is evidence of an association with mortality. As we wanted to ensure the usability of the smartphone app, we sought to create the most parsimonious model with maximal performance based on the combination of the Akaike Information Criterion, AUC, and goodness of fit. In addition to the variables used to construct the original C-Score, we investigated the predictive value of including sociodemographic characteristics such as gender, race or ethnicity, marital status, and educational attainment, as well as simple medical history variables shown to be associated with mortality, such as binary variables *ever diagnosis of high blood pressure* [24,25] and *ever diagnosis with hypercholesterolemia* [26]. Finally, we included interaction terms (C-Score interacting with each of the additional variables) to explore whether a maximally complex model would perform better.

### Statistical Analysis

To validate the original C-Score, we tested the model using the pooled NHANES data. However, as NHANES lacks the reaction time variable, which is one of the variables used to compute the C-Score, we conducted a sensitivity analysis using data from NHANES III, a smaller survey that collected data on reaction time, to measure the marginal effect of the reaction time variable. Following the validation and sensitivity analysis, we incorporated additional variables into the model and investigated their internal and external validity.

### Validating the Original C-Score

For all models, we used a complete case approach, whereby the only participants included were those for whom a risk score based on all risk factors could be computed (ie, for whom there were no missing data on any of the included variables). We pooled NHANES data from 2005 to 2014, which included 6 out of 7 variables included in the original C-Score model (missing reaction time). As the NHANES survey did not have the reaction time variable, all individuals were assumed to have the maximum score for that variable in this validation exercise.

In the complete case analysis, there were 21,015 participants (aged 18-85 years) with complete information on mortality, age, and all metrics included in the C-Score. This population with a wide age range was selected as one would expect to see greater variability in the exposure variables, thus permitting better exploration of the models. Furthermore, to produce estimates with a population similar to that in the Clift et al [17] study, participants aged 40 to 69 years were analyzed separately [17]. The complete case analysis for this age-restricted subsample included 9994 participants. For each prediction model, we assessed the model’s performance by investigating its discrimination—the extent to which it can adequately discriminate between those who will have the discrete event and those who will not—and calibration—the extent to which the observed and predicted probabilities agree [27,28]. The area under the receiver operating characteristic curves (c-statistics) and a design-based goodness-of-fit test for estimating the F-adjusted mean residual test [29] were used to assess discrimination and calibration, respectively [29]. Unlike the original model, we could not use Cox regressions, given that the NHANES data sets are repeated cross-sections and we did not have the benefits of a longitudinal panel to use Cox. Therefore, our model estimates mortality within a 10-year period (time of follow-up for the NHANES mortality link) instead of the survival time.

In all cases, we ran an additional analysis including both the C-Score and the logarithm of age, as performed by Clift et al [17].

### Sensitivity Analysis of the Original C-Score

As the NHANES survey lacks one of the variables used for validation—the reaction time variable—we performed a sensitivity analysis with a different data set. We conducted a sensitivity analysis using data from NHANES III, a survey conducted from 1988 to 1994 containing data for 33,994 people aged ≥2 months, including mortality data, to ascertain the marginal effect of the reaction time variable from the analysis. Owing to the limited number of people with neurobehavioral indicators, we did not impose age limits in this sensitivity analysis.

The NHANES III data set contains the reaction time variable but lacks 2 of the 7 variables included in the risk model (sleep duration and alcohol consumption). The lack of these variables should drive the fit and calibration of the model downward, and therefore, any results in this sensitivity analysis would be conservative. In this sensitivity analysis, we tested the sensitivity of the 5-variable model to the inclusion and exclusion of the reaction time variable. The complete case analysis yielded data from 1440 participants.

All data analyses were performed using Stata 15 (StataCorp), using survey weights to specify the survey and sample design characteristics. In addition, a dummy variable for the survey round was included in the models with pooled data. For all models, *P* values <.05 were regarded as statistically significant.

### Adapting the C-Score to the US Population and Measuring Its Internal and External Validity

We examined the impact of including additional variables on calibration and discrimination [27,28]. We used the area under the receiver operating characteristic curve (AUC), or the c-statistic, to assess the discrimination of the adapted models. We tested both internal and external validities. We used a k-fold cross-validation procedure to assess within-study model validity [30]. We estimated AUC based on 10 random samples (the *test* samples) that were independent of the samples used to train the model (the *training* sample), averaging the AUCs associated with each individual fold and bootstrapping the cross-validated AUCs to obtain 95% CIs. To assess calibration, we used a design-based goodness-of-fit test of logistic regressions, as well as calibration curves developed using locally weighted scatterplot smoothing to compare fitted outcome probabilities with observed outcome probabilities [31]. We also report the Akaike Information Criterion. For the external validation, we assessed the best-performing model using the NHANES III data set.

This study follows the TRIPOD (Transparent Reporting of a multivariable prediction model for Individual Prognosis Or Diagnosis) guidelines for multivariable prediction models [32].

### Ethics Approval

The NHANES survey is approved by the National Center for Health Statistics Institutional Ethics Review Board. Written informed consent was obtained from all adult participants. Ethical approval to conduct this analysis was not required as we used publicly available data. This study was approved by the institutional review board of the Johns Hopkins Bloomberg School of Public Health and was deemed nonhuman subject research (13743).

### Data Availability

The data sets analyzed in this study are publicly available on the NHANES website. The C-Scores are proprietary information but can be provided as restricted data to the reviewers.

## Results

### Validating the Original C-Score

**Figure 1:**
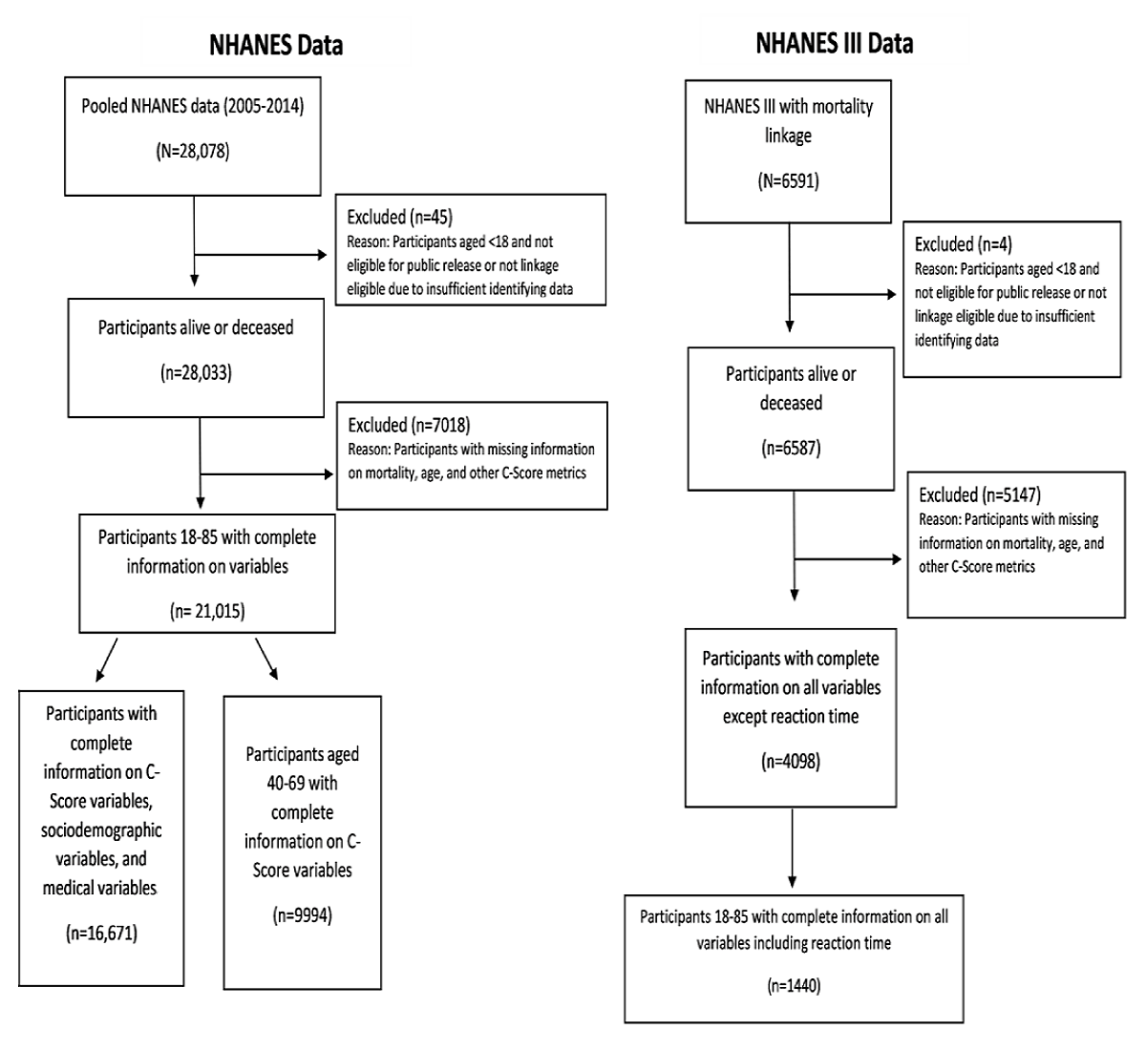
Flowchart for sample sizes for National Health and Nutrition Examination Survey (NHANES) study samples.

From 2005 to 2014, we obtained 28,078 records from the NHANES. Of these, 99.84% (28,033/28,078) were matched with mortality data and 74.84% (21,015/28,078) had complete information on all variables. A flowchart of the sample sizes for the main analysis, sensitivity analysis, and adaptation of the model is shown in Figure 1. The basic characteristics of the study sample are presented in Table 2.

**Table 2:** Descriptive statistics for the different samples used in the study.^a^

Variable		Full study sample (N=21,015)	Age-restricted sample (40-69 years; N=9994)	NHANES^b^ III subsample for the sensitivity analysis (N=1440)
Age (years), mean (SD)		47.43 (17.97)	53.78 (8.53)	47.85 (5.80)
**Sex, n (%)**				
	Male	10,094 (48.03)	4764 (47.67)	655 (45.49)
	Female	10,921 (51.97)	5230 (52.33)	785 (54.51)
**Ethnicity, n (%)**				
	Mexican American	3334 (15.86)	1621 (16.22)	373 (25.9)
	Other Hispanic	1891 (9)	997 (9.98)	36 (2.5)
	Non-Hispanic White	9519 (45.3)	4215 (42.18)	615 (42.71)
	Non-Hispanic Black	4413 (21)	2314 (23.15)	403 (27.99)
	Other race—including multiracial	1858 (8.84)	847 (8.48)	13 (0.9)
Resting heart rate, mean (SD)		72.83 (12.11)	72.42 (11.94)	69.14 (10.80)
Waist to height ratio, mean (SD)		0.59 (0.10)	0.60 (0.09)	0.58 (0.09)
Weekly alcohol intake, mean (SD)		3.63 (8.36)	3.88 (9.07)	N/A^c^
Sleep duration, mean (SD)		6.85 (1.40)	6.73 (1.38)	N/A
**Self-rated health, n (%)**				
	Excellent or very good	8169 (38.87)	3515 (35.17)	558 (38.75)
	Good	8425 (40.09)	4007 (40.09)	538 (37.36)
	Fair	3790 (18.03)	2078 (20.79)	292 (20.28)
	Poor	631 (3)	394 (3.94)	52 (3.61)
Number of cigarettes per day, mean (SD)		3.28 (7.60)	3.94 (8.63)	6.67 (11.71)
Comorbidities, n (%)		3790 (18.41)	2217 (22.27)	183 (12.81)

^a^Survey weights are not included in this descriptive analysis.

^b^NHANES: National Health and Nutrition Examination Survey.

^c^N/A: not applicable.

There were 21,015 participants in the pooled data with complete information on mortality, age, and other C-Score metrics. The mean age of the sample was 47.43 (SD 17.97) years, the mean resting heart rate was 72.83 (SD 12.11) beats per minute, the mean WtHR was 0.59 (SD 0.10), mean weekly alcohol intake was 3.63 (SD 8.63) drinks per week, and mean sleep duration was 6.85 (SD 1.40) hours. For self-rated health, 38.87% (8169/21,015) were *excellent*, 40.09% (8425/21,015) were *good*, 18.03% (3790/21,015) were *fair*, and 3% (631/21,015) were *poor*. There were 48.03% (10,094/21,015) men and 51.97% (10,921/21,015) women. In the study sample, 18.41% (3790/21,015) had existing comorbidities such as diabetes, stroke, coronary heart disease, angina, or heart attack. In terms of the main study outcome, 6.07% (1276/21,015) of patients had died as of December 31, 2015.

In the validation subsample (among participants aged 40-69 years), there were 9994 participants with a mean age of 53.78 (SD 8.53) years, mean resting heart rate of 72.42 (SD 11.94) beats per minute, mean WtHR of 0.60 (SD 0.09), mean weekly alcohol intake of 3.88 (SD 9.07) drinks per week, and mean sleep duration of 6.73 (SD 1.38) hours. For self-rated health, 35.17% (3515/9994) were *excellent*, 40.09% (4007/9994) were *good*, 20.79% (2078/9994) were *fair*, and 3.94% (394/9994) were *poor*. There were 47.67% (4764/9994) of men and 52.33% (5230/9994) of women. In terms of comorbidities, 22.27% (2217 or 22.27%) reported a diagnosis of diabetes, stroke, coronary heart disease, angina, or heart attack. In terms of the study outcome, 95.38% (9532/9994) of participants were alive, and 4.32% (462/9994) had died as of December 31, 2015.

Table 3 shows that in the study sample, higher C-Scores were related to a reduction in the occurrence of all-cause mortality (odds ratio 0.96, *P*<.001, 95% CI 0.95-0.96). The C-Score model showed a good fit for all-cause mortality in this population, with an AUC of approximately 0.72 (95% CI 0.70-0.73). After adding the log of age as a covariate in this model, the calibration test rejected the null hypothesis of good fit; however, the AUC increased to 0.86 (95% CI 0.85-0.87).

**Table 3:** Performance of the C-Score models for all-cause mortality by subsample.^a^

Outcome	C-Score model									C-Score plus log (age)							
	Score OR^b^ (*P* value)	*F*-adjusted test statistic			AUC^c^ (95% CI)		AIC^d^		Score OR (*P* value)			*F*-adjusted test statistic			AUC (95% CI)		AIC
		*F* test (*df*)	*P* value (fit)								*F* test (*df*)		*P* value (fit)				
Full study sample (N=21,015)	0.96 (<.001)	0.52 (9,71)	.86 (good)	0.72 (0.70-0.73)		8897.78		.96 (<.001)			7.25 (9,71)		<.001 (poor)	0.86 (0.85-0.87)		7272.50	
Age-restricted sample (40-69 years; N=9994)	0.95 (<.001)	1.16 (9, 71)	0.34 (good)	0.72 (0.70-0.75)		3458.24		.95 (<.001)			0.50 (9,71)		.87 (good)	0.75 (0.73-0.77)		3366.48	

^a^All models include dummy variables for the survey rounds. Survey weights were included in all analyses.

^b^OR: odds ratio.

^c^AUC: area under the curve.

^d^AIC: Akaike Information Criterion.

Table 3 shows that in the full study sample, the model demonstrated a good fit when not including the logarithm of age. Among the participants aged between 40 to 69 years, C-Score models, both with and without log age, had a good fit for all-cause mortality. Values of AUC ranged between 0.72 (95% CI 0.70-0.75) to 0.75 (95% CI 0.73-0.77).

### Sensitivity Analysis

In the sensitivity analysis, we obtained data from NHANES III (1988-1994) on 6591 participants, of whom 21.85% (1440/6591) had complete data to conduct the validation. Table 4 shows the C-Score model had generally a good fit for all-cause mortality and an AUC of 0.68 (95% CI 0.65-0.72). The addition of reaction time worsened the model fit. The tables show that in the predictive C-Score model without reaction time but with age, all-cause mortality had a good fit, with an AUC of 0.72 (95% CI 0.69-0.75). After adding reaction time, the AUC for all-cause mortality did not differ.

**Table 4:** Sensitivity analysis on all-cause mortality for the marginal effect of the reaction time variable using NHANES^a^ III (N=1440).^b^

Outcome	C-Score model						
	Score OR^c^ (*P* value)	*F*-adjusted test statistic			AUC^d^ (95% CI)		AIC^e^
		*F* test (*df*)	*P* value (fit)				
C-Score model performance with reaction time	0.92 (<.001)	2.97 (9,41)	.01 (poor)	0.68 (0.65-0.72)		1556.57	
C-Score model performance without reaction time	0.91 (<.001)	1.82 (9,41)	.09 (good)	0.68 (0.65-0.72)		1555.48	
C-Score model plus log age performance with reaction time	0.92 (<.001)	0.86 (9,41)	.56 (good)	0.72 (0.69-0.75)		1438.43	
C-Score model plus log age performance without reaction time	0.92 (<.001)	0.97 (9,41)	.48 (good)	0.72 (0.69-0.75)		1485.04	

^a^NHANES: National Health and Nutrition Examination Survey.

^b^All models included a dummy variable for the survey rounds. Survey weights were included in all analyses. The C-Score was calculated using five out of seven covariates: waist to height ratio, self-rated health, resting heart rate, smoking, and reaction time. The C-Score was calculated using 4 out of 7 covariates.

^c^OR: odds ratio.

^d^AUC: area under the curve.

^e^AIC: Akaike Information Criterion.

### Adapting the C-Score to the US Population and Measuring Its Internal and External Validity

#### Overview

Of the 21,015 participants with complete information on the C-Score metrics, 20,626 (98.15%) had information on sociodemographic characteristics and of those, 16,671 (80.82%) had complete information on medical history variables. Thus, the final analytic sample in which the C-Score was adapted comprised 16,671 participants. Table 5 outlines the characteristics of this sample. The average age of the respondents in this sample was 50.43 (SD 17.32) years, and a little more than half (8831/16,671, 52.97%) were female. The mean resting heart rate was 72.53 (SD 12.04) beats per minute, mean WtHR was 0.59 (SD 0.10), mean weekly alcohol intake was 3.32 (SD 7.37) drinks per week, and mean sleep duration at night was 6.84 (SD 1.40) hours. For self-rated health, 39.48% (6581/16,671) reported *excellent health*, 40.09% (6617/16,671) reported *good health*, 18.03% (2948/16,671) reported *fair health*, and 3% (525/16,671) reported *poor health*. Approximately 21.03% (3497/16,671) of the respondents had existing comorbidities such as diabetes, stroke, coronary heart disease, angina, or heart attack. There were 6.3% (1062/16,671) deaths recorded in the analytic sample.

**Table 5:** Characteristics of the research sample (N=16,671).

Variable			Analytical sample
Age (years), mean (SD)			50.43 (17.32)
**Sex, n (%)**			
	Male	7840 (47.03)	
	Female	8831 (52.97)	
**Ethnicity, n (%)**			
	Mexican American	2142 (12.85)	
	Other Hispanic	1447 (8.68)	
	Non-Hispanic White	7944 (47.65)	
	Non-Hispanic Black	3543 (21.25)	
	Other race (including multiracial)	1595 (9.57)	
Resting heart rate (beats per minute), mean (SD)			72.53 (12.04)
Waist to height ratio, mean (SD)			0.59 (0.096)
Weekly alcohol intake (drinks per week), mean (SD)			3.32 (7.37)
Sleep duration (hours per night), mean (SD)			6.84 (1.39)
**Self-rated health, n (%)**			
	Excellent or very good	6581 (39.48)	
	Good	6617 (39.69)	
	Fair	2948 (17.68)	
	Poor	525 (3.15)	
Number of cigarettes per day, mean (SD)			2.97 (7.25)
Comorbidities, n (%)			3497 (21.03)
Deaths, n (%)			1062 (6.3)

The addition of sociodemographic variables and medical history variables (model 3), in contrast, similarly increased the AUC of the original C-Score model from 0.72 to an AUC of 0.87 (95% CI 0.86-0.88), although without a loss in the goodness of fit.

Upon inclusion of interaction terms between each of the covariates and the C-Score variable, we did not obtain significant increases in AUC or fit, indicating that this more complex model does not offer much improvement compared with a more parsimonious model. In addition, the C-Score odds ratio was not significant, implying no change in the odds of all-cause mortality associated with the change in the C-Score.

**Figure 2:**
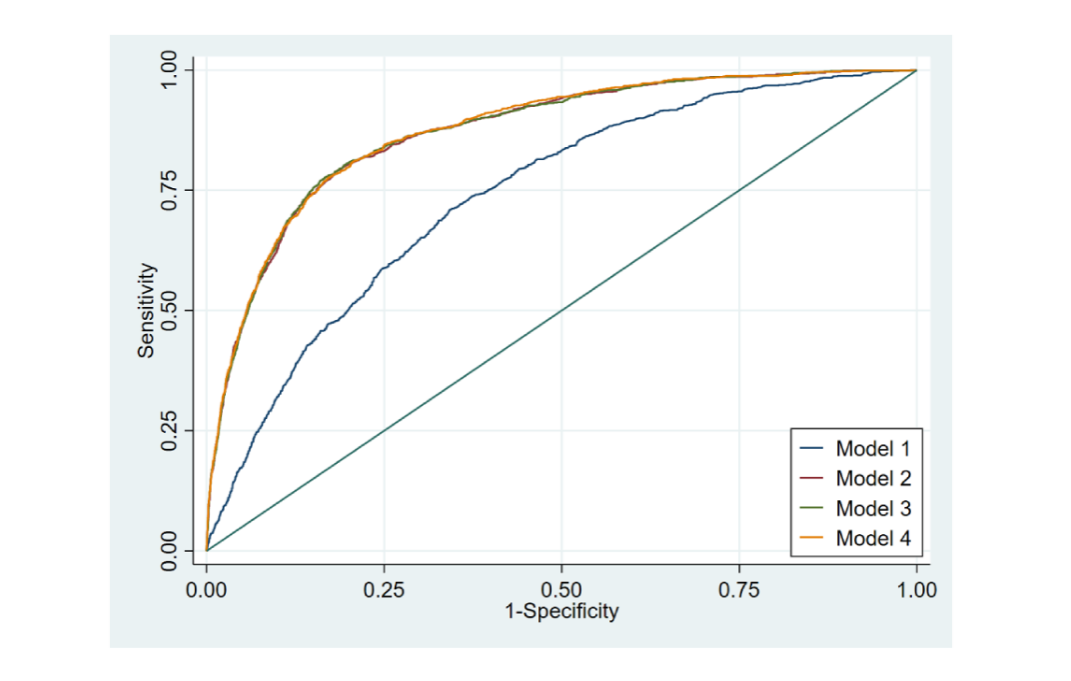
Receiver operating characteristic curve for original C-Score versus expanded models for all-cause mortality. Model 1: C-Score; model 2: C-Score+sociodemographic variables; model 3: C-Score+sociodemographic variables+medical variables; model 4: C-Score+sociodemographic variables+medical history+interactions.

**Figure 3:**
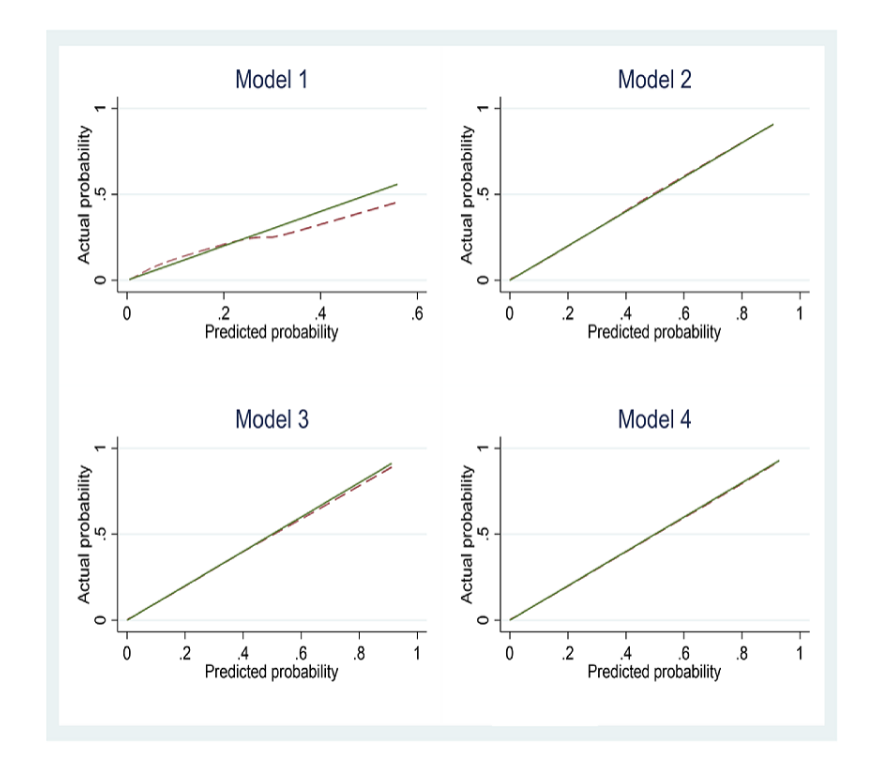
Calibration plots of predicted versus observed probabilities for original C-Score versus expanded models for all-cause mortality. Model 1: C-Score; model 2: C-Score+sociodemographic variables; model 3: C-Score+sociodemographic variables+medical variables; model 4: C-Score+sociodemographic variables+medical history+interactions.

Table 6 and Figures 2 and 3 compare the performance of the expanded models with that of the basic C-Score model. The addition of basic sociodemographic variables to the C-Score model in model 2 increased discrimination considerably, as evidenced by the c-statistic of 0.87 (95% CI 0.85-0.88) compared with 0.72 (95% CI 0.71-0.73) yielded by the original C-Score model. However, although the addition of sociodemographic variables lowered the Akaike Information Criterion, the model was not well calibrated as the calibration test rejected the null hypothesis of good fit (*P*=.04).

**Table 6:** Performance of original C-Score versus expanded models for all-cause mortality.^a^

Model	Independent variables	Participants, N	Score OR^b^ (*P* value)	Goodness of fit (*P* value)	AUC^c^ (95% CI)	AIC^d^
1	C-Score^e^	21,015	0.96 (<.001)	Good fit (.86)	0.72 (0.70-0.73)	8897.78
2	C-Score^e^+sociodemographic variables^f^	20,626	0.97 (<.001)	Poor fit (.04)	0.87 (0.85-0.88)	6977.07
3	C-Score^e^+sociodemographic variables^f^+medical history^g^	16,671	0.96 (<.001)	Good fit (.06)	0.87 (0.86-0.88)	5705.134
4	C-Score^e^+sociodemographic variables^f^+medical history^g^+interactions^h^	16,671	1.0 (.25)	Good fit (.19)	0.87 (0.86-0.89)	5693.319

^a^All models include dummy variables for the survey rounds. Survey weights were included in all analyses.

^b^OR: odds ratio.

^c^AUC: area under the curve.

^d^AIC: Akaike Information Criterion.

^e^C-Score included six variables: cigarette consumption, alcohol consumption, sleep duration, self-rated health, waist to height ratio, and resting heart rate.

^f^Sociodemographic variables included age, gender, race or ethnicity, marital status, and educational attainment.

^g^Medical history variables were *ever diagnosis of high blood pressure* and *ever diagnosis with hypercholesterolemia*.

^h^Each sociodemographic variable and medical history variable interacted with the C-Score.

#### Internal Validation

**Figure 4:**
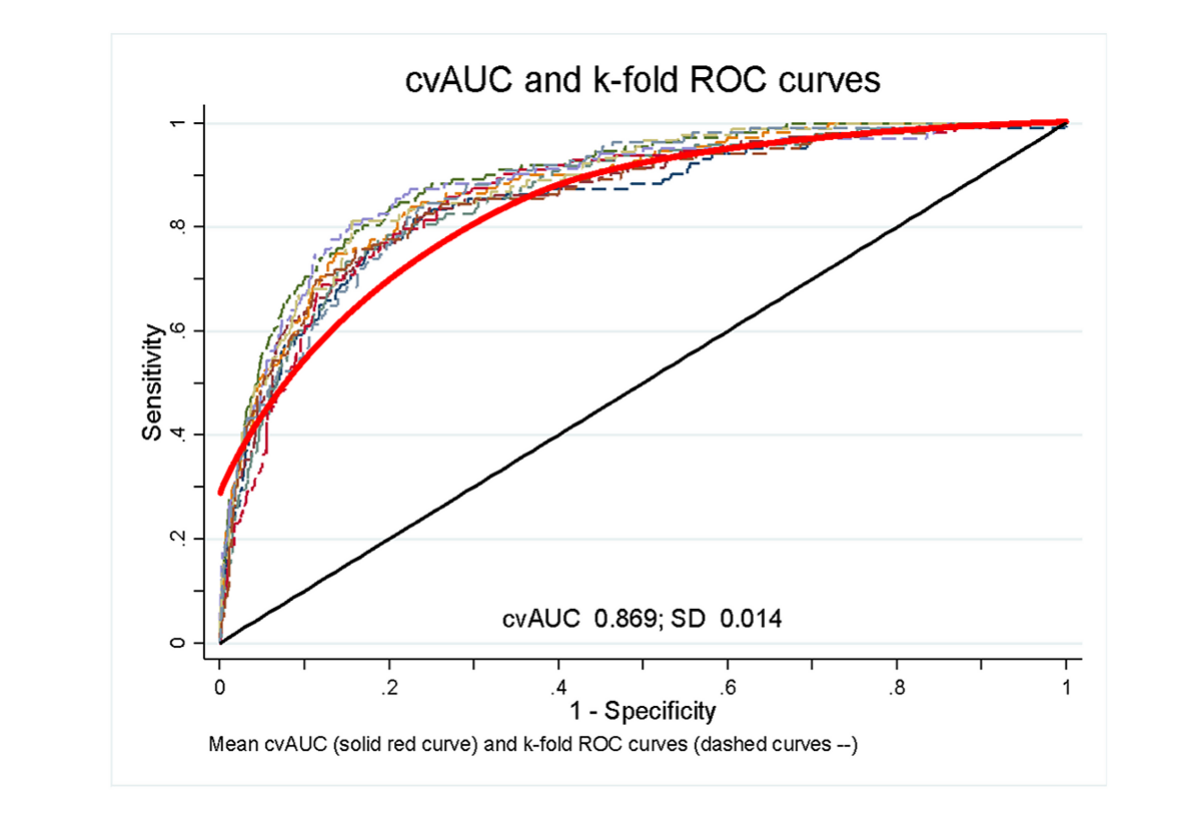
Internal validation using k-fold procedure (folds=10). cvAUC: cross-validation area under the curve; ROC: receiver operating characteristic.

The validity of our final model (model 3) was assessed using k-fold cross-validation. We used 10 random samples to determine the discrimination capability of the model in predicting the future incidence of all-cause mortality. The AUCs for these random samples ranged from 0.85 to 0.87, showing high consistency in the discrimination of the model (Figure 4). The mean cross-validation AUC was 0.869, indicating a strong capability of the model to discriminate the incidence of all-cause mortality.

#### External Validation

**Figure 5:**
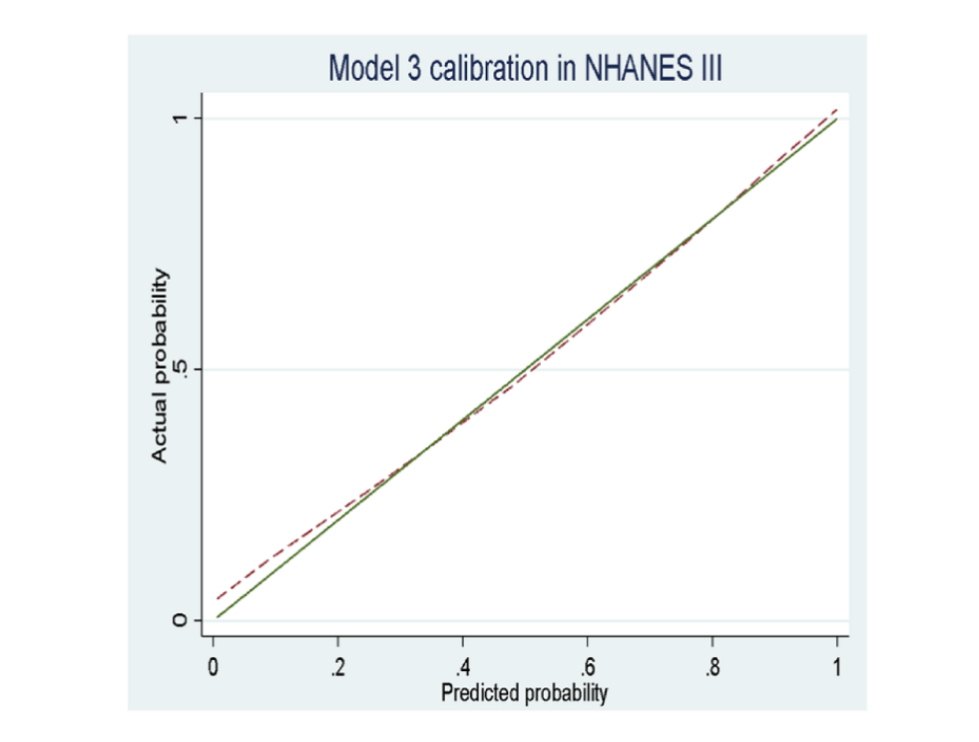
Calibration plot of predicted versus observed probabilities of all-cause mortality for model 3 for all-cause mortality. Model 3: C-Score+sociodemographic variables+medical variables. NHANES: National Health and Nutrition Examination Survey.

The best-performing model (model 3) of the main analysis was used for external validation. Figure 5 shows a calibration plot displaying the predicted versus observed probabilities of all-cause mortality. A comparison between the model performance in the research sample and the external validation sample reveals that the C-score using NHANES 2005-2014 has a good fit with *P*=.06, AUC of 0.87 (95% CI 0.86-0.88) and an Akaike Information criteria of 5705.13. The C-score on the NHANES III survey has a good fit with *P*=.45, AUC of 0.89 (95% CI 0.88-0.90) and an Akaike Information criteria of 3420.19. These results imply that the model performed very well in the external validation sample. It was both well-calibrated and had a high AUC, which is even higher than that identified in the first sample.

## Discussion

### Principal Findings

In this study, we conducted external validation of the C-Score in the US population and expanded the original score to improve its predictive capabilities in the US population.

We found that the C-Score had generally good prediction and calibration capabilities and that it is a promising model that could provide fast and accurate information on all-cause mortality through a digital health app. Our results reveal similar AUCs compared with those found in the United Kingdom by Clift et al [17].

Given the lack of the reaction time variable in the main NHANES sample, we conducted a sensitivity analysis with another survey (NHANES III), which contains the reaction time variable, to assess its marginal effect in predicting all-cause mortality. The results suggest that the absence of the reaction time variable did not meaningfully change the calibration or the discrimination attributes of the assessed model. We believe that the marginal effect is likely to be low as part of the variance explained by the reaction time variable might be captured by other variables in the C-Score.

In addition, we showed that the incorporation of a set of basic sociodemographic and medical history variables greatly boosted the model’s predictive performance in the US general population. The AUC for our final model greatly increased from 0.72 (95% CI 0.71-0.73) for the basic C-Score model to 0.87 (95% CI 0.86-0.88) in the expanded model. We further assessed the internal and external validity of the expanded model and found that the model performed equally well in the 10-fold cross-validation sample and the external NHANES III data set.

The incorporation of this model into a user-friendly digital health app can motivate users to predict their current and future health status and take actions to modify their health, thus potentially shaping their future trajectories. Consumer demand for technological innovations that measure health status and predict health outcomes is evidenced by the recent proliferation in the use of commercial wearable technologies, ranging from simple activity or exercise monitors to more sophisticated home-based connected medical devices [4,5]. These devices may function independently or leverage sophisticated back end analytics to analyze user trends and provide feedback [33]. In addition to catering to consumer demand for quick, robust, and user-friendly health assessment, these digital health strategies also engage health care providers by sending client-generated data directly into electronic health records, enabling their integration into care plans [34,35]. The past decade has seen a clear increase in obesity and other chronic diseases worldwide, especially in the US population, where cardiovascular disease, cancer, chronic respiratory illness, and diabetes are leading causes of death and morbidity [36]. An increasing proportion of adults and children worldwide are overweight or obese, exacerbating the risk of future noncommunicable diseases (NCDs) [37]. The availability of scores that can help individuals reliably estimate current (and potentially future) risk of adverse outcomes could be helpful in interventions to improve individual and, thus, population health in the United States and worldwide. Thus, our validation of the C-Score serves to validate a promising predictive model that can be easily accessed by a lay audience to predict individualized clinical risk and take action to make beneficial lifestyle changes and consequently reduce the risk of future adverse outcomes.

Recent evidence confirms the utility of wearable technology in predicting clinical outcomes with high accuracy [4,38]. Previous studies have capitalized on wearable technologies to provide reliable and accurate measurements of established predictors of mortality and adverse health outcomes [39-42]. For example, Smirnova et al [42] found that wearable technologies provide reproducible and unbiased measures of physical activity, which, in turn, outperform traditional predictors of 5-year mortality among older adults in the US population [42]. The adapted C-Score model had the added strength of using variables that are routinely captured in baseline data collected from users of wearables or inpatient records maintained by health care systems. In addition, such data are more uniformly measured and available across different settings outside the United States and the United Kingdom. Given the overall goal of increasing the generalizability of this score, this is a step in the right direction toward making this a more universally feasible model. Previous models that leveraged complete blood counts and metabolic profiles achieved similar performance (AUC 0.83-0.90) at a presumably much higher cost and logistical complexity [43]. Other studies that integrated a wide range of cognitive, demographic, lifestyle, and clinical factors also achieved similar, if not lower, performance. For example, Ajnakina et al [44] achieved an AUC of 0.74 for all-cause mortality prediction in the general population using 13 prognostic factors. Models that apply increasingly more complex methods such as machine learning are able to slightly improve discrimination, yielding AUCs between 0.78 and 0.79 [45].

Our findings should be viewed in light of some limitations. First, we used a cross-sectional survey that did not follow individuals over time. NHANES is the only survey that is nationally representative of the US general population, which contains most of the variables present in the original C-Score model. The NHANES survey contains 6 out of the 7 variables included in the original UK population-based model, potentially leading to a C-Score that artificially underperforms when predicting all-cause mortality. However, our sensitivity analysis showed that the reaction time variable did not marginally provide additional value to the C-Score in this sample. Even if the subsample in which we tested the reaction time variable did not have the external validity to inform the results of the NHANES subsample, the lack of the reaction time variable would likely lead to an underperforming score, implying that the ability of the score to predict all-cause mortality would be higher, if the reaction time variable had been available in the main NHANES data set. Moreover, although the association between death and other covariates has been investigated using Cox proportional hazards models in other publications, including the original C-Score model [45,46]—we focused on a binary all-cause mortality variable instead of time to death as (1) time to event data was not available, (2) logistic models are easier to communicate to a lay audience, and (3) they avoid the assumptions made by Cox models that may not be met [42]. They have also been shown to perform as well as more complex models [42,47]. Ideally, we would have preferred to use a data set that provides longitudinal estimates; however, we used NHANES, a cross-sectional survey, as it is the only US survey that is nationally representative of the general population and contains the variables present in the original C-Score model (with the exception of reaction time). It also provides a large data set with population-based data.

### Conclusions

Limitations notwithstanding, the findings of this validation indicate that the performance of the C-Score is fairly good for predicting all-cause mortality in the US population. The adapted risk score had even better prediction capabilities, as evidenced by the finding that it predicted 87% of the mortality in the US population.

In conclusion, our study findings validate and expand a novel risk-scoring algorithm that can predict the risk of all-cause mortality among adults in the general population with high accuracy and which could be incorporated into a digital health application. The use of high-performing risk scores could be instrumental in clinical counseling, choice of care pathways, and even patient-driven behavior change interventions targeting modifying lifestyles and promoting behavioral change. Despite known effective strategies to reduce NCD-related deaths worldwide, chronic and preventable NCDs continue to drive adult mortality. High-performing risk scores that trigger behavior change could be instrumental in stemming this tide of death and decreased global productivity.

## Abbreviations

AUC: area under the curve
NCD: noncommunicable disease
NDI: National Death Index
NHANES: National Health and Nutrition Examination Survey
TRIPOD: Transparent Reporting of a multivariable prediction model for Individual Prognosis Or Diagnosis
WtHR: weight to height ratio
